# Increasing return-to-work among people on sick leave due to common mental disorders: design of a cluster-randomized controlled trial of a problem-solving intervention versus care-as-usual conducted in the Swedish primary health care system (PROSA)

**DOI:** 10.1186/s12889-018-5816-8

**Published:** 2018-07-18

**Authors:** Elisabeth Björk Brämberg, Kristina Holmgren, Ute Bültmann, Hanna Gyllensten, Jan Hagberg, Lars Sandman, Gunnar Bergström

**Affiliations:** 1 Unit of Intervention and Implementation Research for Worker Health, Institute of Environmental Medicine, Karolinska Institutet, 171 77 Stockholm, Sweden; 2 Department of Public Health and Community Medicine, Sahlgrenska Academy, University of Gothenburg, Box 414, 405 30 Göteborg, Sweden; 3 Närhälsan, Region of Västra Götaland, Lillhagsparken 6, 442 50 Hisings-Backa, Sweden; 4 Department of Health and Rehabilitation, Sahlgrenska Academy, University of Gothenburg, Box 455, 405 30 Göteborg, Sweden; 5 Department of Health Sciences, University Medical Center Groningen, Community and Occupational Medicine, Groningen, the Netherlands; 6 Division of Insurance Medicine, Department of Clinical Neuroscience, Karolinska Institutet, 171 77 Stockholm, Sweden; 7 Institute of Health and Care Sciences, Sahlgrenska Academy, University of Gothenburg, Box 457, 405 30 Gothenburg, Sweden; 8 National Centre for Priorities in Health, Department of Medical and Health Sciences, Linköping University, 583 81 Linköping, Sweden; 9 Centre for Musculoskeletal Research, Department of Occupational and Public Health Sciences, University of Gävle, 801 76 Gävle, Sweden

**Keywords:** Common mental disorders, Adjustment disorders, Anxiety disorders, Depression, Cluster-randomized trial, Problem solving skills, Return to work, Sick leave, Economic evaluation, Ethical evaluation

## Abstract

**Background:**

Common mental disorders affect about one-third of the European working-age population and are one of the leading causes of sick leave in Sweden and other OECD countries. Besides the individual suffering, the costs for society are high. This paper describes the design of a study to evaluate a work-related, problem-solving intervention provided at primary health care centers for employees on sick leave due to common mental disorders.

**Methods:**

The study has a two-armed cluster randomized design in which the participating rehabilitation coordinators are randomized into delivering the intervention or providing care-as-usual. Employees on sick leave due to common mental disorders will be recruited by an independent research assistant. The intervention aims to improve the employee’s return-to-work process by identifying problems perceived as hindering return-to-work and finding solutions. The rehabilitation coordinator facilitates a participatory approach, in which the employee and the employer together identify obstacles and solutions in relation to the work situation. The primary outcome is total number of sick leave days during the 18-month follow-up after inclusion. A long-term follow-up at 36 months is planned. Secondary outcomes are short-term sick leave (min. 2 weeks and max. 12 weeks), psychological symptoms, work ability, presenteeism and health related quality of life assessed at baseline, 6 and 12-month follow-up. Intervention fidelity, reach, dose delivered and dose received will be examined in a process evaluation. An economic evaluation will put health-related quality of life and sick leave in relation to costs from the perspectives of society and health care services. A parallel ethical evaluation will focus on the interventions consequences for patient autonomy, privacy, equality, fairness and professional ethos and integrity.

**Discussion:**

The study is a pragmatic trial which will include analyses of the intervention’s effectiveness, and a process evaluation in primary health care settings. Methodological strengths and challenges are discussed, such as the risk of selection bias, contamination and detection bias. If the intervention shows promising results for return-to-work, the prospects are good for implementing the intervention in routine primary health care.

**Trial registration:**

ClinicalTrials.gov Identifier: NCT03346395 Registered January, 12 2018.

## Background

It is estimated that common mental disorders (CMDs), i.e. mild to moderate depression, anxiety, adjustment and stress-related disorders affect about one-third of the European working-age population. In Sweden, after a reduction in sickness absence due to CMDs between 2005 and 2010, sickness absence started to increase again in 2011. At present, CMDs are the most common cause of sick leave in Sweden. In January 2016, CMDs were the cause of about 45% of all sick leave among women and 32% of all sick leave among men [1]. Besides the individual suffering, with its negative impact on the individual’s well-being, financial circumstances, social network and risk of stigmatization, the cost for society in terms of health care, sick leave and productivity loss for employers is high. In 2013, the total costs for mental health in Sweden, including sick leave, amounted to some SEK 65 billion (approx. EUR 6 billion) [2].

The first choice of clinical treatment for CMDs is psychological treatment, primarily cognitive behavioral therapy and/or medication [3]. These treatments have positive effects on symptom reduction and improved functioning, yet symptom reduction is not accompanied by increased return-to-work (RTW) [4, 5]. Previous research shows that, to increase RTW among persons on sick leave due to CMDs, work place interventions which include cooperation between the person on sick leave, his/her employer, the health care services, the social insurance agency and the occupational health services are needed [6–8]. Cooperation between the stakeholders (i.e. health care services, employers, social insurance agency, and occupational health services) is a prerequisite for reducing sick leave and increasing RTW. However, a major problem is the lack of cooperation between these stakeholders. Moreover, primary health care often fails to focus on RTW and workplace related interventions [9, 10].

In line with previous research, we found that the first concern of primary health care professionals is patients’ psychological and physical well-being; the process of RTW is often of secondary importance [10]. On the basis of previous evaluations of an evidence-based intervention (i.e. interventions based on problem-solving therapy), we know that this type of intervention has promising effects on sick leave among persons with CMDs [7, 11]. However, the Swedish primary health care system has a limited history of workplace interventions which include cooperation with the workplace. This represents a new challenge for primary health care.

Workplace interventions are defined as interventions which target or involve the workplace, work organization, work conditions or work environment and/or occupational (case) management strategies with active stakeholder involvement [12]. Evidence of effective workplace RTW interventions for people with CMDs is limited and the studies vary with respect to inclusion criteria (cf. [13, 14]). However, it has been shown that interventions with a workplace component, including vocational counseling, are more likely to succeed in increasing RTW than interventions that do not include such a component (cf. [6, 15, 16]). The British National Institute of Clinical Excellence’s guideline on long-term sickness absence and work incapacity identifies early, multidisciplinary and workplace-focused interventions as those most likely to give positive results [17]. In a recent review, no significant benefits were found for coordinated RTW programs [13]. However, only two of the review’s 14 studies reported outcomes for populations with CMDs and these were conducted outside Sweden, in coutries with other social security systems than the Swedish system [13]. A key element of workplace interventions is cooperation between the employer, the person on sick leave and health care professionals. The cooperation between these actors in the RTW process has been described as challenging because each actor represents different perspectives and interests [9].

Previous studies have shown that problem-solving skills and work-focused cognitive behavioral therapy are more effective than guideline-based interventions in reducing the time to first RTW among employees on sick-leave for CMDs. Guideline-based interventions did not reduce time to return to full-time work [7, 14, 18, 19]. In a web-based intervention which included problem-solving skills and relapse prevention, a significantly faster first RTW was found for the intervention group. No significant differences were found for time until full RTW or in reduced total number of days on sick leave at the 12-month follow-up [20]. A review of interventions to improve time to first RTW among people with depression gave moderately good evidence that workplace interventions in combination with clinical interventions reduced the number of days on sick leave significantly more than clinical interventions alone [21]. Similar results were found by an updated review which evaluated the effects of workplace interventions among workers with mental health problems. The results demonstrated a significantly better reduction in time to first RTW than care-as-usual [15]. A cluster-randomized controlled trial in the Netherlands demonstrated the 12-month effectiveness of a problem-solving intervention for reducing recurrent sick leave among workers with CMDs. The intervention group had a lower incidence of recurrent sick leave than the control group which received care-as-usual [11]. Economic evaluations of such interventions have shown varying results, from reduced to increased health care costs [22–25].

To date, only a limited number of studies have evaluated the effectiveness of interventions to improve RTW in a primary health care population [6, 7, 15]. There is thus a need for more RTW research in the primary health care context. Previous studies of problem-solving interventions have been conducted in countries with work environments, sickness absence and social security systems which differ from those in Sweden [6, 7, 11, 15]. The (financial) implications of employers to cooperating with the health care services may also differ in the Swedish system. These differences may in turn affect the effective components of the intervention and influence the way the intervention works and how likely it is to result in successful outcomes. Most studies have primarily focused on time to first RTW [6, 15] or recurrent sick leave after RTW [7]. The effect evaluations have been conducted in occupational health service settings, have had relatively small sample sizes and lacked a long-term follow up (i.e. ˃ 12 months) [8]. In Sweden, the primary health care service is responsible for treatment, issuing sickness certificates and assessing the individual’s work capacity. Hence, it is important to adapt the problem-solving intervention to the primary health care setting and evaluate the intervention in that context.

This paper presents the design of a study, which evaluates a work-related problem-solving intervention provided at primary health care centers (PCCs) for employees on sick leave due to common mental disorders. We hypothesize that participants who have undergone the problem-solving intervention will have fewer total sick leave days and fewer recurrent sick leave periods after RTW than the participants who receive care-as-usual (CAU). In addition, a process evaluation of the intervention and its association with the effects on sick leave, an economic evaluation and an evaluation of the ethical issues raised by the intervention will be conducted.

## Methods

The CONSORT statement and the extension for randomized controlled trials were used to describe the study design [26, 27].

### Study design and setting

**Fig. 1:**
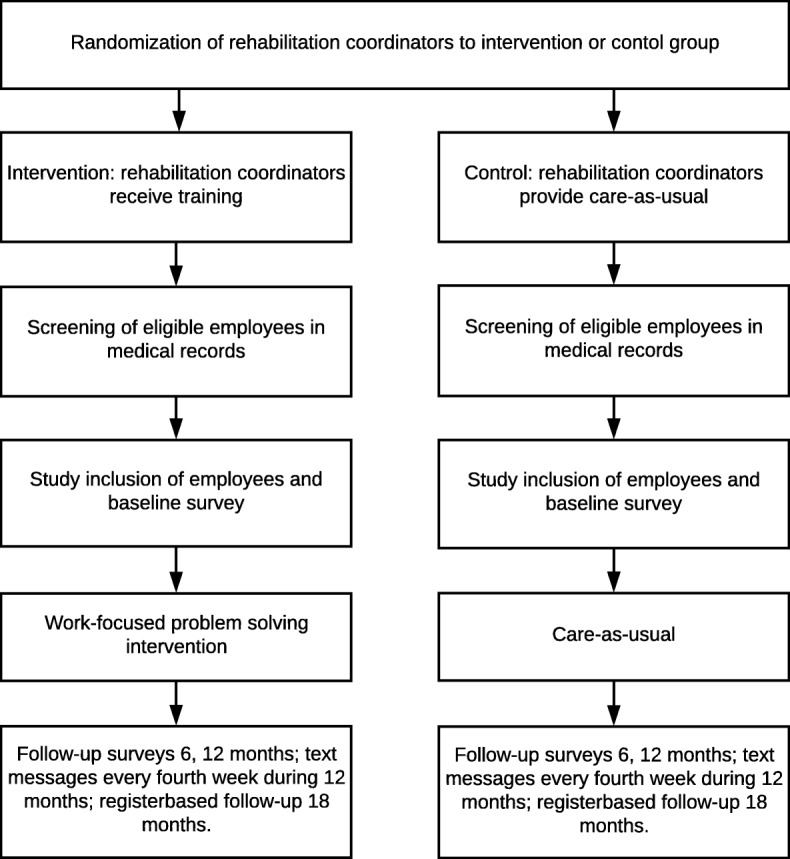
Flowchart and overview of the trial

The study is designed as a two-armed cluster-randomized controlled trial of a problem-solving intervention to reduce sick leave compared with CAU (Fig. 1). In Sweden, persons who cannot work due to disease or injury are entitled to benefits from a mainly tax-funded social insurance system. Individuals who are gainfully employed receive economic compensation from their employer for the first 14 days (except for one qualification day). Thereafter, the Social Insurance Agency grants the benefits. A sickness certificate issued by a physician in the primary health care system is needed from day 8. In an effort to improve RTW, Swedish county councils have introduced rehabilitation coordinators (RCs) whose role is to coordinate the RTW of persons who are on sick leave for whatever reason. RCs are commonly registered nurses, physiotherapists, occupational therapists or social workers.

The study will be conducted in the primary health care service of the Västra Götaland region of Sweden. This service comprises some 200 publicly-funded PCCs which provide health care for 1.6 million people of all ages. Around 180 (90%) of the 200 PCCs have an on-site RC.

### Recruitment of rehabilitation coordinators

RCs were recruited from PCCs in the Västra Götaland region. Information about the study was sent to managers in the region by the first author (EBB) and was given at meetings with PCC managers and RCs. Only RCs not participating in other ongoing interventional studies at the time of inclusion were invited to participate. RCs were also excluded if they had an upcoming leave of absence (for example pregnancy leave, a sabbatical) or if they were leaving the PCC or retiring. About 80 coordinators received an invitation. Written information about the study was provided as well as a registration form and informed consent. Written informed consent was obtained from all RCs. The recruitment process resulted in 19 RCs covering 24 PCCs.

### Recruitment of participants

To be eligible for the study an employee must be on sick leave due to CMDs and must have been diagnosed by a physician at a PCC which is participating in the study. The current sick leave period should last a minimum of 2 weeks and a maximum of 12 weeks. Employees on sick leave will be recruited by a research assistant who screens medical records for inclusion and exclusion criteria. The research assistant will be blinded to group assignment. Employees who meet the inclusion criteria will be invited to join the study by the research assistant by email. Eligible employees will receive written information about the study. Those employees willing to participate must give written consent. They will then be sent the baseline questionnaire by email.

### Eligibility criteria

#### Inclusion criteria

Eligible participants can be included if they meet the following criteria:

- Employed women and men aged 18–59 years
- On sick leave (i.e. a minimum of 2 weeks and a maximum of 12 weeks) with mild to moderate depression, anxiety or adjustment disorder (F 32, F 41, F 43) as the primary reason for sick leave, diagnosed by a physician according to the Swedish version of international statistical classification of diseases and related health problems - tenth revision
- Physician works at a PCC in the Västra Götaland region
- Accept the employer’s involvement
- Understand written and spoken Swedish.

All eligible participants who meet the criteria and consent to participation will be included.

#### Exclusion criteria

Employees with severe depression; other severe mental disorders (i.e. psychotic or bipolar disorders or who have been referred to a psychiatrist); pregnancy; somatic complaints or disorders that affect work ability; those unable to read, write and understand Swedish will be excluded.

### Intervention

#### Training of rehabilitation coordinators in the intervention group

The RCs will take part in a two-day training course about the problem-solving intervention [28]. The training is provided by a licensed psychologist with extensive experience of problem-solving therapy and of supervising primary health care professionals. The RCs will complete a questionnaire about the quality of the training course and their problem-solving skills before and after the course, as a part of the process evaluation. The RCs will have support from a manual and three work sheets developed by the research group, all based on research into CMDs, the problem-solving intervention and the coordinator function. During the intervention period the RCs will receive supervision and feedback from the project leader whenever needed.

#### The problem-solving intervention

The intervention focuses on the individual and the involvement of the workplace [11, 29] in addition to CAU [3]. The added value on top of CAU is the intervention’s problem-solving process and the coordination by the RC between the employee on sick leave, his/her employer and the health care professionals.

**Fig. 2:**
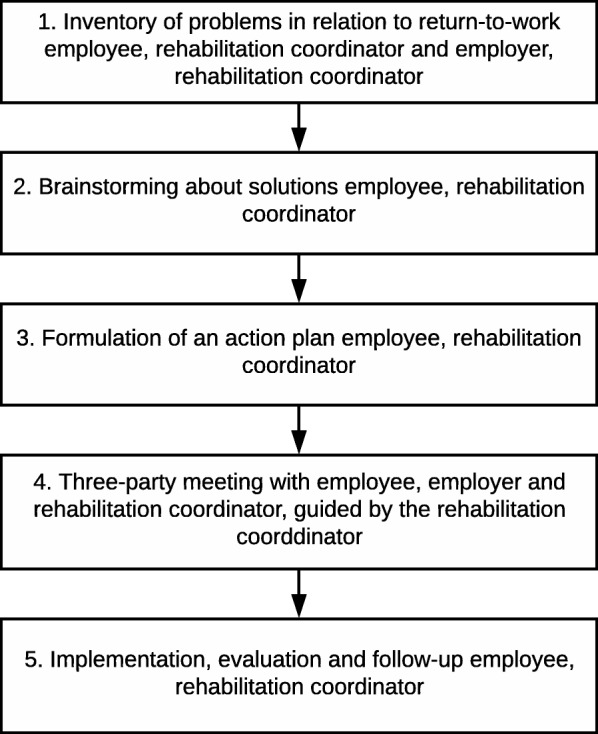
Overview of the problem solving intervention

Communication between the employee, the RC and the employer is needed for making an inventory of problems, opportunities and identifying the help needed to solve the problems [11]. The present intervention is a modified version of the Dutch sharp-at-work intervention [29] which aimed to prevent recurrence of sick leave among employees with CMDs who had returned to work. The Dutch target group differed from the target group of the present study, i.e. persons on sick leave. The new, adapted intervention is a five-step problem-solving process which aims to identify and find possible solutions to problems perceived as obstructing the RTW process (Fig. 2). A meeting with the person on sick leave, his/her employer and the RC is included in the process, as previous research has stressed the importance of communication between the different stakeholders [30]. The intervention comprises the following five steps:

1. Making an inventory of problems and/or opportunities related to RTW; a collaborative approach involving employee and RC; employer and RC.
2. Brainstorming about solutions, involving employee and RC.
3. Writing down solutions, identifying the support needed to implement them and assessing their applicability, involving employee and RC.
4. A meeting with the person on sick leave, the employer and the RC to discuss solutions and make an action plan, involving employee, RC and employer.
5. Evaluation of the action plan and implementation of solutions, involving employee and RC.

The work-focused problem-solving intervention starts with the RC interviewing the employee who is on sick leave. The RC facilitates the employee’s participation in his/her RTW-process by supporting and encouraging the employee to take active part in brainstorming and prioritizing obstacles and possible solutions for RTW. The first consultation between the employee and the RC lasts about 45 min and is summarized in the form of a preliminary list of identified problems and possible solutions. Thereafter the RC interviews the employer by telephone about problem orientation, e.g. how aware the employer is of the employee’s health problems; how these problems are apparent in the workplace; the employer’s understanding of the causes of the illness. This takes approximately 15 min. The next step is a meeting of 45–60 min between the employee, the employer and the RC. In the problem-solving process, the RC facilitates a dialogue between the employee and the employer; thus the employee and employer are responsible for identifying obstacles and facilitators related to RTW. They also identify and prioritize solutions and make an action plan. The action plan also addresses relapse prevention. The RC evaluates the action plan with the employee.

### Care as usual (CAU)

The RCs in the control condition will continue to deliver CAU during the study period. CAU may include strategies for the RTW process and may involve the employer. However, the strategies used by the RCs to date are not structured in the same way as in the intervention condition, and do not follow an explicitly stated guidance by problem-solving therapy.

CAU involves cognitive behavioral therapy or medical treatment, or a combination of both [3], as well as the coordination of RTW (meeting with a RC). We will make no specific attempts to ensure that the physicians follow the guidelines for the treatment of CMDs [3]. The participants, their physicians and, if relevant, their psychologists, will not receive any information from the project group about the problem-solving intervention.

### Measurements and procedure

Data will be collected by questionnaires, text messages and the use of registers. Register data will be collected from the Micro Data for the Analysis of Social Insurance register (MiDAS) from the Social Insurance Agency, the Västra Götaland region’s Vega register which is the patient register for Region Västra Götaland, and the Longitudinal Integration Database for Health Insurance and Labor Market Studies (LISA), administrated by Statistics Sweden. Web-based questionnaires will be administered at baseline and after 6 and 12 months. Text messages will also be sent to the employees every fourth week over a 12-month period. The use of text messages enables us to measure short-term sick leave (≤ 14 days) that is not covered by the MiDAS register. A 36-month follow-up of sickness absenteeism based on register data is planned.

### Primary outcome

#### Registered sick leave

The primary outcome is the total number of days of sick leave 18 months after baseline. Data from MiDAS will be collected from baseline until the 18-month follow-up and for the 24 months before baseline. Information about the number of days on sick leave, episodes of sick leave after RTW, and receipt of disability pension will be collected for all participants. Sickness benefit and disability pension will be analyzed separately.

The total number of days of registered sick leave will be calculated after 36 months in the same way as at the 18-month follow-up.

#### Secondary outcomes

Self-reported short-term sick leave, part-time registered sick leave, psychological symptoms, work ability, impairment of work performance, presenteeism, health-related quality of life (HRQoL).

##### Self-reported sick leave and return to work

Short-term sick leave will be assessed by the question: ‘How many days in the past four weeks have you been absent from work because of illness? Answer with a number between 0 and 28 days’. Self-reported, short-term sick leave has been shown to demonstrate acceptable reliability [31]. The time from baseline until 1) partial RTW or 2) full RTW (defined as working ordinary hours for an uninterrupted period of at least four weeks) will be calculated. The prevalence of no sick leave, partial and full-time sick leave during the 12-month follow-up will be calculated.

##### Part-time registered sick leave

Part-time registered sick leave will be calculated from baseline during the 18-month follow-up as registered part-time (25/50/75% of full-time) sickness absenteeism.

##### Psychological symptoms

Reduction of self-reported anxiety and depression will be measured by the Hospital Anxiety and Depression Scale, a well-established and much used scale in primary health care with 14 items used for the assessment of anxiety (7 items) and depression (7 items) [32]. The response format is a 4-point Likert scale, ranging from 0 to 3, with higher scores indicating anxiety and/or depression. Self-reported, stress-related exhaustion disorder will be assessed by means of the self-reported exhaustion scale (s-ED), a scale which has demonstrated construct and predictive validity. The response format is yes/no [33].

##### Work ability

Work ability will be measured as improved working ability and reduction in work-related stress. The measure is self-reported work ability assessed by three items from the Work Ability Index. The items concern perceived work ability in relation to physical and mental demands at work and the employee’s prognosis of his/her work ability two years hence [34].

##### Sickness presenteeism and work performance

Sickness presenteeism will be measured by the single question: ‘Did you, in the last six months, go to work even though you felt that you really should have taken sick leave because of your state of health?’ [35]. The response format is a 4-point scale ranging from 0 (never) to 4 (more than 5 times). This item has been extensively used in previous research [35, 36]. In addition, two items about impairment of work performance due to 1) health problems (presenteeism) and 2) work-environment problems will be assessed. The first has been developed and adapted from the Work Productivity Impairment – General Health Questionnaire [37]; the second has been developed and tested for validity and reliability in Sweden [38]. The response format for both is 0 to 10, with higher scores indicating that health problems or work-environment problems have prevented the employee from working.

##### Health problems

HRQoL will be assessed by the EuroQoL health state questionnaire (EQ-5D). The response format is a 3-level scale, with higher levels indicating severity [39]. We will use the subscale for insomnia from the Karolinska Sleep Questionnaire, to assess sleeping problems. The response format range from 0 (never) to 5 (always), higher scores indicate severity [40].

##### Prognostic measures

A variety of prognostic measures for sick leave will be included in the study. At baseline, participants’ personal characteristics (e.g. age, gender, marital status, educational level), workplace characteristics (e.g. sector, profession) and sick leave two years before inclusion will be collected from the LISA and MiDAS registers. In addition, questions covering all dimensions from the demand-control-support model [41] will be included as a prognostic variable. Psychological and social factors at work and work-family balance will be assessed by items from the General Nordic Questionnaire [42] which focus on ongoing conflicts with the employer/superior, perceived loss of control over work tasks, conflicts between the employee’s values and how work is carried out and work-family balance. Organizational factors at work will be assessed by items from the Copenhagen Psychosocial Questionnaire [43] with focus on the emotional demands made by the employee’s work. Finally, the employee’s self-efficacy in RTW will be assessed as a prognostic factor [44].

Information about treatment (i.e. psychological counseling, number of sessions, use of antidepressants) and health care consumption will be collected from employees’ medical records and the Vega register from baseline until the 18-month follow-up.

### Process evaluation

As recommended by Moore GF*,* et al. [45], a process evaluation will be conducted. The process evaluation [46] will examine (1) the study’s fidelity, i.e. whether and to what extent it is possible to implement the intervention according to the protocol, (2) reach, dose delivered and dose received, i.e. the relationship between the key elements of the intervention and the effect outcome, and (3) the study’s context, i.e. how the RCs, the persons on sick leave, their employers, their physicians and other stakeholders (e.g. occupational health service, social insurance agency officer) perceive the intervention and the obstacles and enablers which influence the implementation. Process-evaluation data will be collected by means of questionnaires, employees’ medical records and individual interviews with RCs, employees and their employers.

*Fidelity* will be measured by a questionnaire containing questions about whether the intervention was delivered as planned. The RCs in the intervention group will report overall adherence to the manual/fidelity to the method for each employee during the study, after the employee has participated in the problem-solving process. *Reach* is measured for RCs and employees. The RCs participation in the two-day training course will be assessed by attendance and their satisfaction with the course. Eligibility will be screened by medical records, while reach will be assessed as those who are willing to participate in the study. *Dose delivered* will be assessed by means of a questionnaire for the RCs which asks questions about the frequency of face-to-face meetings with the employee, number of telephone follow-ups and the frequency and quality of the three-party meetings with the employer. *Dose received* is defined as the whether or not the participants receive the problem-solving intervention. Dose received will be measured by how much of the intervention that the participants received; number of sessions with the RC; how much the participants were exposed to the different components of the intervention. The content and fulfillment of the recommended follow-ups with the employees will be addressed. The RCs in the control group will receive a check list which helps them to report the content in the control condition.

### Economic evaluation

A cost-utility analysis will be conducted to analyze changes in HRQoL measured by EQ-5D and translated using the Swedish experience-based value set [39] and societal costs. Direct costs for health care will be estimated based on administrative data from the Vega register. Indirect costs will be estimated using the human capital approach from sick leave (self-reported and registered in MiDAS) and self-reported presenteeism. All-cause health care use will be collected from the Vega register over 18 months after baseline. This register covers diagnoses, the reason for visits and measures taken for patients visiting primary and specialized healthcare in the Västra Götaland region of Sweden [47].

Moreover, a cost-effectiveness analysis will be conducted to compare changes in days on sick leave to the direct health care costs, including the costs for conducting the intervention, calculated based on time reported by the RCs.

### Evaluation of ethical issues

An ethical analysis will be performed focusing on central ethical values and norms for the Swedish health care system: patient autonomy and privacy, equality and fairness as well as professional ethos and integrity. The ethical analysis will analyse whether the changes brought about through the intervention will emphasise or mitigate ethical challenges in relation to this patient group. Data will be collected through focus groups interviews with the different stakeholders: employees, employers, rehabilitation coordinators, physicians responsible for sickness certification, social insurance agency officers and representatives of occupational health services. Interview guides based on an ethics framework used in health-technology assessment will be developed [48]. Focus group interviews will be analysed using manifest content analysis.

Based on the results from the focus groups interview and taking into considerations the actual changes implied by the intervention, strategies for how to handle identified ethical challenges or problems will be developed. The methodology of reflective equilibrium will be used, where strategies are developed to be consistent with established ethical values and norms within the Swedish health care system, as found in the Swedish health care legislation and in health care practice [49–51].

#### Randomization

The study has a two-armed cluster randomized design and takes place at the level of RCs. The RCs are randomized into the intervention group or the control group (CAU) by means of computer-generated random numbers. The randomization is stratified according to the PCCs care-need index. This index includes variables such as age, education, employment status, single household and single parent for all persons belonging to each PCC. The index is expected to have an impact on the results and consequently a balance within the strata is required.

A RC covers between one and three PCCs. The RCs in the Västra Götaland region have their own networks and meetings four times a year. Because it is important to avoid contamination, the RCs allocated to the intervention arm are instructed to talk about the intervention only with each other.

#### Blinding

This study has a cluster-randomization design, in which the employees follow the RCs at the PCCs and are randomized before they give informed consent. The employees in both the intervention and the control group receive the same information about the study to equalize employees’ expectations about their participation in the study. The employees are blinded for the treatment allocation since they receive no information about the other group. The RCs are instructed not to tell employees that the study consists of two groups, to ensure that the employees are not aware about the two conditions. The RCs are not blinded for treatment allocation because the RCs in the intervention arm will know that they will receive education and training. An independent statistician will perform the analyses and will be blinded for participant allocation.

#### Data analysis

Statistical analyses adapted for cluster-randomized controlled trials will be conducted. Linear and generalized linear models will be used. Intention-to-treat analyses and, if relevant, per-protocol analyses will be conducted. Potential confounders will be adjusted for in the analyses, if they prove to be unevenly distributed and might be expected to have an impact on the outcomes when the intervention is compared with the control condition. Possible interaction effects on the primary and secondary outcomes for (1) gender x treatment, and (2) number of sessions with RC x treatment will be assessed. If they are statistically significant, appropriate analyses will be considered. Data collected from interviews and focus groups will be analyzed by methods adapted for qualitative data and normative analysis (cf. [48, 52, 53]).

##### Statistical power

We estimate that the study population needs to be 220 participants: 110 participants in the intervention and the control group respectively. The participants will be obtained by sampling 10 clusters with 11 participants each in the intervention group and the same number of clusters and participants in the control group. With these numbers it will be possible to achieve around 80% power to detect a difference between the groups of at least 20% of registered sickness absence 18 months after baseline. The intra-cluster correlation was set to 0.010. This sample size calculation is relevant for statistical analyses that take into account a correction factor for the effect of a clustered design and use an alpha level of 0.05.

## Discussion

The study is designed as a pragmatic, effectiveness trial that will investigate whether employees on sick leave will benefit from a problem-solving intervention delivered by RCs in primary health care. Analyses will be performed to examine whether the problem-solving intervention has been more successful in reducing sick leave than CAU. In a parallel process evaluation, the RCs’ adherence to the protocol and how treatment fidelity, dose delivered and dose received might influence the sickness absence outcome will be investigated. Furthermore, the process evaluation will increase our understanding of the obstacles to and facilitators of the intervention, contextual factors and the experience of taking part in the intervention. The economic evaluation will examine the intervention’s economic impact on society and the health care system that is expected to deliver the intervention if it is successful. In a health care system guided by and founded on ethical values and norms, it will be essential to relate to, and have strategies for how to handle identified ethical challenges. This knowledge will form the basis for future implementation of the problem-solving intervention in primary health care.

### Methodological considerations

The cluster-randomization, in which the RCs are randomized to intervention and control groups, reduces the risk of contaminating the employees who participate in the study. The RCs will only provide the problem-solving intervention or CAU. Another strength of the study is that participants will be selected by a project assistant who screens medical records for eligible employees on sick leave. The project assistant will have no previous knowledge about the participants or the randomization, thereby reducing the risk of selection bias.

In the Swedish social insurance system, the employer usually pays for sick leave up to and including day 14. The MiDAS register therefore contains sick leave data from day 15 of sick leave. Since short-term sick leave may predict future long-term sick leave, valuable information about short-term sick leave will be collected from the employees. However, with self-reported questionnaires there is always a risk of recall bias and drop-out. The use of text messages facilitates repeated measurements with short recall periods, while the questionnaires are administered at baseline and at 6 and 12-month follow-up. The 6 and 12-month follow-ups may increase the risk of recall bias, but this risk has to be weighed against the frequency of the questionnaires that the participants have to fill in.

The data collected in the process evaluation will be analyzed before the primary and secondary outcomes in the effectiveness evaluation are known to the research group, to prevent biasing interpretations of the intervention’s effectiveness. One of the strengths of the proposed method for the economic evaluation will be its use of comprehensive individual-level data collected for administrative purposes, on both direct and indirect costs. A strength of the proposed methodology for ethical analysis is it context-sensitivity in relation to the Swedish health care system and its inherent ethical values and norms. However, ethical analysis is, on top of demands for internal consistency, dependent upon being critically examined and acknowledged as relevant by the stakeholders. Hence, dialogue concerning this analysis is crucial.

### Possible impact of results

Sick leave due to CMDs is a matter of national and international concern, not only for the individuals themselves but also for their employers and society at large. The costs of sick leave for society are high. Consequently, finding interventions that reduce these costs as well as the human suffering represents cost effectiveness for society and employers and greater well-being for individuals. Moreover, previous economic evaluations of the problem-solving intervention have shown ambiguous results with regards to its cost/effect balance. Costs are furthermore known to differ between countries, based on e.g. the financing of the health care systems. Thus, there is a need for more thorough examination of the intervention’s economic impact. Improved work ability and worker health will be beneficial for individuals, their employers, enterprises, organizations and society at large. The ethical analysis will identify ethical challenges and obstacles but also ethical advantages with the intervention, and thereby provide basis to assess how the intervention relates to the value basis of the Swedish health care and welfare system. In a health care system, where new interventions are constantly introduced, such analyses are essential to understand how the intervention fits into these value systems. If the intervention shows promising results for RTW and work ability, there are good prospects for being able to implement and disseminate it in the primary health care service, which may result in positive effects for society at large (e.g. lower costs for sick leave and rehabilitation).

## Abbreviations

CAU: Care-as-usual
CMDs: Common mental disorders
HRQoL: Health-related quality of life
LISA: Longitudinal integration database for health insurance and labor market studies
MiDAS Micro: Data for the Analysis of Social Insurance
PCC: Primary health care center
RC: Rehabilitation coordinator
RTW: Return-to-work
